# Postoperative Morbidity After Radical Resection of Retroperitoneal Solitary Fibrous Tumor

**DOI:** 10.3389/fsurg.2022.833296

**Published:** 2022-03-28

**Authors:** Aobo Zhuang, Yuan Fang, Lijie Ma, Weiqi Lu, Hanxing Tong, Yong Zhang

**Affiliations:** ^1^Department of General Surgery, South Hospital of the Zhongshan Hospital/Shanghai Public Health Clinical Center, Fudan University, Shanghai, China; ^2^Department of General Surgery, Zhongshan Hospital, Fudan University, Shanghai, China

**Keywords:** retroperitoneal, solitary fibrous tumor, neoadjuvant radiotherapy, preoperative embolization, morbidity, progression-free survival (PFS), overall survival (OS)

## Abstract

**Background:**

This study aimed to investigate the clinicopathological characteristics of retroperitoneal solitary fibrous tumor (RSFT) and the safety of radical resection.

**Methods:**

A retrospective analysis was conducted on the data of 32 RSFT patients who received surgery with curative intent from February 2011 and June 2021.

**Results:**

This cohort included 16 (50%) male and 16 (50%) female patients, with the median age of 52 (29 to 72) years. Tumor burden ranged from 3 to 25 (median, 10) cm. Seven patients received arterial embolization before surgery. 15 (47%) patients received radiotherapy, nine (28%) of which received preoperative radiotherapy. Most of the patients (91%) achieved complete resection with median bleeding of 400 (20 to 5,000) ml. Nine (28%) patients received packed red blood cell (RBC) transfusion, with a median of 5 (2 to 10) U. All patients had the five-year progression-free survival rate and the overall survival rate of 75.8% and 80.0%, respectively. 11 (34%) patients were found with adverse events, and four (12%) patients were found with serious postoperative complications (Clavien-Dindo ≥3), of which one (3.1%) patient died after surgery. The univariate analysis found that tumor burden (*p* = 0.022), packed RBC transfusion (*p* = 0.001) and postoperative hospital stays (0.027) were correlated with overall morbidity. The multivariate analysis found packed RBC transfusion as an independent risk factor for postoperative morbidity (HR 381.652, 95% CI, 1.597–91213.029, *p* = 0.033).

**Conclusion:**

RSFT was confirmed as an uncommon, slow-growing and recurring tumor, with acceptable postoperative morbidity and mortality after surgical resection.

## Introduction

Solitary fibrous tumor (SFT), previously named as hemangiopericytoma, has been found as a rare tumor derived from the mesenchyme. The incidence rate of SFT reaches nearly 0.2 per 100,000 population per year (1). Klemperer et al. first described SFT as a pleural tumor, and it was later found on the external thorax (e.g., adrenal glands, head and neck, retroperitoneum, kidney, liver and skeletal muscle) (2). Surgical resection is recognized as the cornerstone of treatment, which can make the optimal long-term prognosis of SFT. The recurrence-free survival rate of SFT is >90% with complete resection (3, 4), while large SFTs are often highly vascular (5). Besides, the retroperitoneum is a huge anatomical space, which often has a greater tumor burden compared with other parts. Furthermore, the scope of resection is generally limited by the surrounding structures (6).

There have been rare studies on retroperitoneal SFT (RSFT), with only two literature reports previously. One literature report is conducted by Rahul Rajeev et al., which contained 18 RSFT patients (7), and the other is a report of 35 primary RSFT patients by Peng Luo et al. (8), whereas relevant postoperative complications data were not included.

This study aimed to investigate the clinicopathological features of RSFT and the safety of radical resection.

## Methods

This retrospective study was conducted at the South Hospital of Zhongshan Hospital/Shanghai Public Health Clinical Center. As approved by the ethics committee, this study retrospectively analyzed the clinicopathological data of patients pathologically diagnosed with SFT (or hemangiopericytoma) from February 2011 to June 2021. Further selection criteria included (1) the location at retroperitoneum, (2) for radical surgery, and (3) complete clinicopathological data and available follow-up information.

The data included gender, age, ASA score, symptoms, presentation status (primary or recurrence), tumor location (abdominal or pelvic), multifocality, tumor burden, malignancy, Ki-67, preoperative embolization, radiation, neoadjuvant radiotherapy, chemotherapy, complete resection, estimated blood loss, packed RBC transfusion, postoperative ICU stay, postoperative complications [Clavien-Dindo classification (9)], as well as postoperative hospital stays.

Tumor burden referred to the sum of the largest diameters of all tumors reported in the surgical record. Complete resection was defined as negative margins (R0) or positive micro margins (R1) without positive gross margin resection (R2). Malignant SFT was diagnosed for lesions with at least one of the characteristics below: hypercellularity, mitotic index> 4/10 at high power field, necrosis, margin of infiltration, as well as pleomorphism (10). Severe postoperative adverse events were classified as Clavien-Dindo 3 or higher.

For the postoperative follow-up, the respective follow-up required clinical and imaging examination (CT or MRI of the chest, abdomen and pelvis). Patients were followed up every 3–4 months for the first 2 years, every 6 months after 2 years, as well as every year after 5 years. Information acquired during follow-up involved disease progression and death. Imaging findings of new lesions or significant enlargement of the original lesions were defined as disease progression.

## Results

### Baseline Characteristics

**Figure 1:**
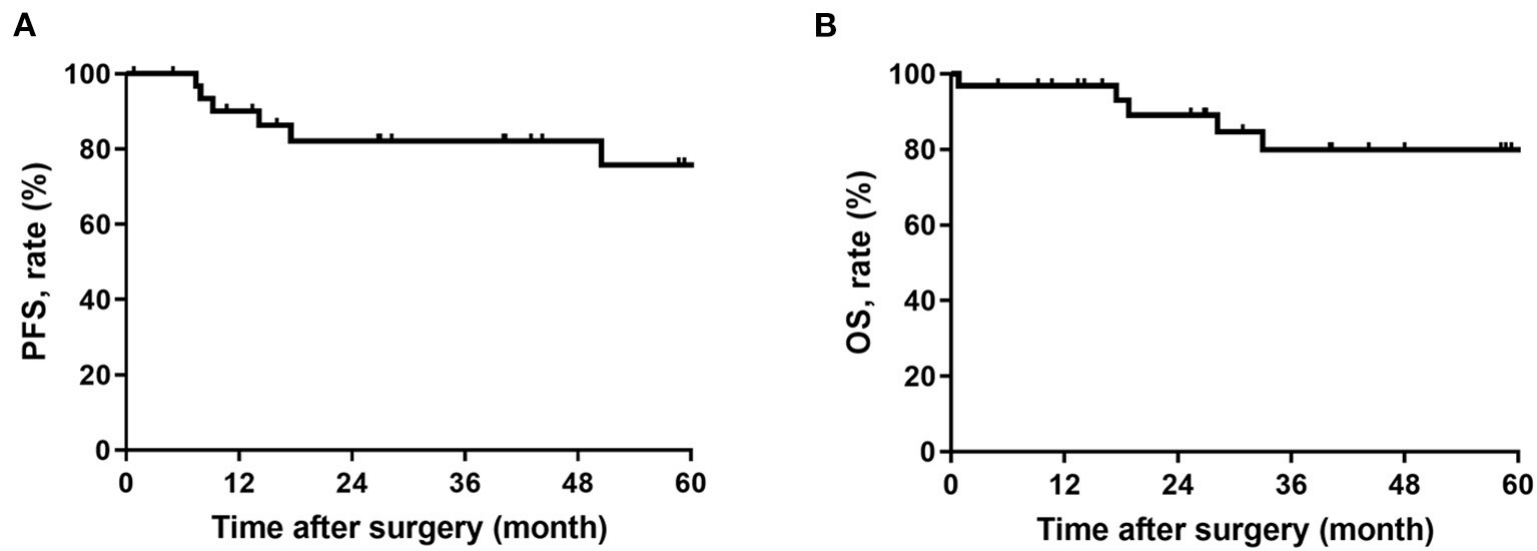
**(A)** Progression-free survival in patients with retroperitoneal solitary fibrous tumor; **(B)** Overall survival in patients with retroperitoneal solitary fibrous tumor.

This study included 32 RSFT patients who received surgical resection with curative intent at South Hospital of Zhongshan Hospital/Shanghai Public Health Clinical Center, of which the details and the summary of clinicopathologic characteristics are listed in Table 1. This cohort covered 16 (50%) male and 16 (50%) female patients, and the median age of all patients was 52 (29 to 72) years. There were eight (25%) patients with ASA classification over grade 1 and seven (22%) patients with recurrent disease. The tumors of most of the patients were located in the pelvic cavity (75%), and with monofocality disease (91%). Tumor burden ranged from 3 to 25 (median, 10) cm. Moreover, 12 (38%) patients were classified to be malignant, while 20 (62%) patients were classified to be benign. Seven patients had undergone arterial embolization before surgery with the median bleeding of 500 (range, 20–3,500) ml. A total of 15 (47%) patients received radiotherapy, nine (28%) patients of which received preoperative radiotherapy. Most patients achieved complete resection (91%), with median bleeding of 400 (range, 20–5,000) ml. Nine (28%) patients received packed RBC transfusion, with a median infusion of 5 (range, 2–10) U. At the median follow-up time of 58.2 (95%CI, 36.1-80.4) months, six (19%) patients had disease recurrence, and five (16%) patients died. The 5-year disease-free survival rate and the overall survival rate of all patients reached 75.8% and 80.0%, respectively (Figure 1).

**Table 1:** Patient and tumor characteristics in 32 patients with retroperitoneal solitary fibrous tumor.

**Characteristics**	***N* = 32**	**% of Total**	**Complication group**	**Non-complication group**	***P*-value**
Gender					0.458
Male	16	50	4	12	
Female	16	50	7	9	
Age, years median (range)	52 (29–72)		51 (30–70)	52 (29–72)	0.802
ASA score					0.397
1	24	75	7	17	
≥1	8	25	4	4	
Symptoms					0.703
Yes	8	25	2	6	
No	24	75	9	15	
Primary disease					1.000
Yes	25	78	9	16	
No	7	22	2	5	
Location					0.397
Abdominal cavity	8	25	4	4	
Pelvic cavity	24	75	7	17	
Multi-focal disease					1.000
Yes	3	9	1	2	
No	29	91	10	19	
Tumor burden, cm median (range)	10 (3–25)		15 (5–25)	7 (3–20)	0012
Histologic subtypes					0.465
Benign	20	62	8	12	
Malignant	12	38	3	9	
Ki-67	5 (1–40)		5 (2–20)	5 (1–40)	0.346
Preoperative embolization					0.667
Yes	7	22	3	4	
No	25	78	8	17	
Radiation	15	47	5	10	0.907
Yes	15	47	5	10	
No	17	53	6	11	
Neoadjuvant radiotherapy					0.681
Yes	9	28	4	5	
No	23	72	7	16	
Chemotherapy					0.111
Yse	2	6	2	0	
No	30	94	9	21	
Complete resection					1.000
Yes	29	91	10	19	
No	3	9	1	2	
Number of combined resections					
0	16	50	5	11	
1	12	38	3	9	
2	2	6	1	1	
3	0	0	0	0	
4	2	6	2	0	
Estimated blood loss, ml median (range)	400 (20–5,000)		600 (50–5,000)	250 (20–2,200)	0.011
Packed RBC transfusion					<0.001
Yes	9	28	8	1	
No	23	72	3	20	
Packed RBC transfusion, unit median (range)	5 (2–10)		6 (6–6)		0.777
ICU Stay					0.730
Yes	10	31	4	6	
No	22	69	7	15	
Clavien–Dindo classification					0.206
NA	21	66	0	21	
1–2	7	22	7	0	
3–5	4	12	4	0	
Postoperative hospital stay, days median (range)	12 (4–55)		20 (8–55)	11 (4–30)	
Disease recurrence					0.637
Yes	6	19	1	5	
No	26	81	10	16	

11 (34%) patients were found with adverse events, and four (12%) were found with severe postoperative adverse events. The results showed that one (3.1%) patient died after surgery, one (3.1%) suffered bleeding, one (3.1%) had urinary tract infection, two (6.3%) had sepsis, two (6.3%) had wound infection, two (6.3%) had intraabdominal abscesses, and two (6.3%) had ascites.

### Risk Factor Analysis

In the univariate analysis, the probability of patients with a postoperative adverse event increased significantly with tumor burden (*p* = 0.022), packed RBC transfusion (*p* = 0.001) and postoperative hospital stays (0.027). The variables with *p* < 0.2 (i.e., tumor burden, number of combined resections, estimated blood loss, packed RBC transfusion, as well as postoperative hospital stays) according to the univariate analysis were applied for the multivariate analysis. In the multivariate logistic analysis, only packed RBC transfusion was still statistically significant (HR 381.652, 95% CI, 1.597–91213.029, *p* = 0.033) (Table 2).

**Table 2:** Univariable and multivariable analyses to determine independent predictors of adverse events of retroperitoneal solitary fibrous tumor.

**Variables**	**Univariate analysis**		**Multivariate analysis**	
	**Hazard ratio (95%CI)**	***P*-value**	**Hazard ratio (95%CI)**	***P*-value**
Gender female vs. male	2.333 (0.520–10.478)	0.269		
Age (continuous)	1.008 (0.951–1.068)	0.794		
ASA score >1 vs. 1	2.429 (0.470–12.542)	0.289		
Symptoms yes vs. no	1.429 (0.303–6.737)	0.652		
Primary disease yes vs. no	1.406 (0.225–8.783)	0.715		
Location Abdominal vs. Pelvic	2.429 (0.470–12.542)	0.289		
Multi-focal disease yes vs. no	0.950 (0.076–11.803)	0.968		
Tumor burden (continuous)	1.188 (1.025–1.377)	0.022	1.144 (0.897–1.459)	0.277
Histologic subtypes Malignant vs. Benign	0.500 (0.103–2.436)	0.391		
Ki-67 (continuous)	0.960 (0.883–1.044)	0.342		
Preoperative embolization yes vs. no	1.594 (0.286–8.871)	0.595		
Neoadjuvant radiotherapy yes vs. no	1.829 (0.374–8.937)	0.456		
Complete resection yes vs. no	0.975 (0.277–3.436)	0.968		
Number of combined resections (continuous)	1.882 (0.845–4.194)	0.122	1.504 (0.370–6.112)	0.569
Estimated blood loss (continuous)	1.001 (1.000–1.002)	0.073	0.999 (0.996–1.001)	0.316
Packed RBC transfusion yes vs. no	5.333 (4.804–592.100)	0.001	381.652 (1.597–91213.029)	0.033
ICU stay yes vs. no	1.429 (0.303–6.737)	0.652		

## Discussion

SFT has been found as a rare, slow-growing neoplasm of mesenchymal tissue origin, which largely occurs in middle-aged patients (3, 11). The incidence of ESFT is significantly higher than that of pleural counterpart, taking up nearly 70% of all SFTs (2), and retroperitoneum is one of the most common sites of ESFT (4, 12). Surgical resection remains the cornerstone of a radical cure (3). However, since SFT is a highly vascularized tumor, there has been no lack of case reports of intraoperative hemorrhage and even death from intraoperative hemorrhage (13–15). Moreover, since RPS often grows huge and requires multiple organ resections, the perioperative risk is elevated (16). Accordingly, the perioperative safety of RSFT should be verified urgently.

There have been only two cohort reports on RSFT in the past. Peng Luo et al. studied 35 patients with retroperitoneal SFT and reported that tumor size ≥10 cm was independently correlated with short DFS. Compared with our cohort, the two groups had comparable malignant ratios (37 vs. 38%) and median tumor sizes (9 vs. 10 cm), but no one was treated with radiotherapy in Peng's cohort, and the postoperative morbidity and mortality of the patients were not reported (8). Another study was reported by Rahul Rajeev et al. (7) in 2015 by complying with The National Cancer Database data. Since the number of patients is limited (only 18 patients), postoperative complications were not systematically reported or analyzed. This is the first study to assess the postoperative morbidity and mortality of RSFT. In this study, adverse events were reported in 11 (34%) patients, four (12%) patients suffered from a serious complication after surgery, one (3.1%) patient died after surgery. The short-term prognosis of the patients in this study complied with the results reported in previous studies on RPS (17–19). For instance, Tseng et al. (17)used data from the American College of Surgeons National Surgical Quality Improvement Program (ACS-NSQIP) to study the safety of radical RPS resection. In the ACS-NSQIP study, the results of the entire cohort included a mortality rate of 1.3%, a morbidity rate of 26%, as well as a severe morbidity rate of 11.5%. Therefore, as indicated by the findings from our sarcoma center, RSFT radical resection is relatively safe.

In this study, transfusion requirements were the significant predictors of postoperative adverse events. In particular, nine (28%) patients received packed RBC transfusion in the perioperative period, and eight (89%) of them were found to develop postoperative complications. This proportion was only 13% who did not receive packed RBC transfusion, consistent with the results of two recent reports from the Transatlantic Australasian RPS Working Group (TARPSWG) on the perioperative safety of primary RPS and recurrent RPS (18, 19). For critically ill patients with massive blood loss or anemia, blood transfusion was found as a life-saving method. In patients who received major surgery, infusion of concentrated red blood cells, fresh frozen plasma, platelets or cryoprecipitate was found to be essential to maintain hemostasis, correct abnormal coagulation, and ensure adequate tissue perfusion and oxygenation (20). However, it has been extensively found that for patients who received surgery, whether to infuse RBC in the perioperative period is correlated with short-term and long-term prognoses (20–22). According to the relationship between perioperative blood transfusion and postoperative systemic inflammatory response, the interference of blood transfusion on postoperative immune disorders could be the mechanism leading to poor prognosis (23). Therefore, strict standards should be formulated based on the evaluation of the appropriateness of blood transfusion, and it is recommended to limit non-essential blood transfusion during the perioperative period.

Neoadjuvant radiotherapy can be used to shrink tumors, which has been found to improve local symptoms and facilitate surgical resection. Targeted radiotherapy of 50 to 60 Gy can decrease the tumor volume from pelvic or thoracic tumors by up to 60% (24–26). Radiation therapy has become one of the main treatments or adjuvant therapy for head, neck and chest SFT. (27, 28). The results of this study showed that there was no additional morbidity or mortality correlated with radiation therapy, consistent with the EORTC STRASS trial, in which the second mid-term safety analysis reported that adjuvant radiotherapy did not increase the risk of perioperative complications (29).

Arterial embolization blocks the terminal arterial supply of tumors, leading to hypoxia and ischemia. It has been employed to effectively treat blood-rich tumors. Since a huge SFT usually has a large blood supply vessel, it has been reported that the blood loss during surgery was large (13), so preoperative selective embolization of the supply vessel of SFTs could reduce intraoperative blood loss without blood transfusion (30, 31). In this study, seven patients received preoperative arterial embolization, and preoperative embolization was not found as a risk factor for postoperative complications in the risk factor analysis. Due to the possibility of intestinal ischemia, especially for tumors with main blood supply as the inferior mesenteric artery, surgery should be performed in 1–2 days after embolization, and patients' abdominal signs should be carefully observed after embolization, so as not to ignore intestinal ischemia complication.

The univariate analysis found that the tumor size was correlated with postoperative complications, while there was no statistical difference in the multivariate analysis. Numerous researchers have reported that the tumor size is negatively correlated with the prognosis of ESFT. Demicco EG et al. and Smith SC et al. suggested that patients with large tumors had decreased DSS (32). Peng Luo et al. also highlighted that tumor size ≥10 cm could be conducive to the independent prognosis of shortened DFS (8). More advanced and larger tumors may lead to longer operation times, greater difficulty and greater inflammation and blood transfusion requirements, thus resulting in the increase in morbidity and mortality.

This study has certain limitations. First, it was a retrospective study, and there were still biases in statistical analysis. Furthermore, though the blood transfusion was found as a risk factor for postoperative disease, the 95% CI range was relatively large due to the limited sample size, so we should draw a careful conclusion. In addition, since only two patients in this study were treated with neoadjuvant chemotherapy and only nine patients received radiotherapy before surgery, it would be difficult to accurately evaluate the efficacy of adjuvant therapy strategies.

This was the first study that presented the clinicopathologic features and analyzed the postoperative morbidity factors for RSFT by using the data of 32 patients from a tertiary cancer center.

SFT refers to a blood-rich tumor, and retroperitoneal tumors are often huge, whereas the overall postoperative safety is controllable, and preoperative radiotherapy and interventional embolization are all possible methods to consider. Intraoperative packed RBC transfusion has been found as an independent risk factor for postoperative complications. We should fine-tune the operation to reduce intraoperative bleeding and reduce RBC transfusion.

## Data Availability Statement

The raw data supporting the conclusions of this article will be made available by the authors, without undue reservation.

## Ethics Statement

The studies involving human participants were reviewed and approved by Ethics Committee of South Hospital of Zhongshan Hospital/Shanghai Public Health Clinical Center. The patients/participants provided their written informed consent to participate in this study. Written informed consent was obtained from the individual(s) for the publication of any potentially identifiable images or data included in this article.

## Author Contributions

AZ, LM, and YF collected, analyzed, and interpreted the patient data. AZ was a major contributor in writing the manuscript. WL provided writing ideas, helped data analysis, and article proofreading. HT and YZ provided the research ideas and guidance and were responsible for the results of this study. All authors contributed to the article and approved the submitted version.

## Conflict of Interest

The authors declare that the research was conducted in the absence of any commercial or financial relationships that could be construed as a potential conflict of interest.

## Publisher's Note

All claims expressed in this article are solely those of the authors and do not necessarily represent those of their affiliated organizations, or those of the publisher, the editors and the reviewers. Any product that may be evaluated in this article, or claim that may be made by its manufacturer, is not guaranteed or endorsed by the publisher.

## References

[1] Vaz Salgado M Soto M Reguero M Muñoz G Cabañero A Gallego I. Clinical behavior of solitary fibrous tumor: a retrospective review of 30 patients. Clin Transl Oncol. (2017) 19:357–63 10.1007/s12094-016-1536-7 27604423

[2] Ronchi A Cozzolino I Marino FZ Accardo M Montella M Panarese I. Extrapleural solitary fibrous tumor: a distinct entity from pleural solitary fibrous tumor. An update on clinical, molecular and diagnostic features. Ann. Diagn. Pathol. (2018) 34:142–50 10.1016/j.anndiagpath.2018.01.004 29660566

[3] England DM Hochholzer L McCarthy MJ. Localized benign and malignant fibrous tumors of the pleura. A clinicopathologic review of 223 cases. Am. J. Surg. Pathol. (1989) 13:640–58 10.1097/00000478-198908000-00003 2665534

[4] Cardillo G Carbone L Carleo F Masala N Graziano P Bray A. Solitary fibrous tumors of the pleura: an analysis of 110 patients treated in a single institution. Ann. Thorac. Surg. (2009) 88:1632–7 10.1016/j.athoracsur.2009.07.026 19853123

[5] Weiss B Horton DA. Preoperative embolization of a massive solitary fibrous tumor of the pleura. Ann. Thorac. Surg. (2002) 73:983–5. 10.1016/s0003-4975(01)03117-4 11899221

[6] Gronchi A Strauss DC Miceli R Bonvalot S Swallow CJ Hohenberger P. Variability in patterns of recurrence after resection of primary retroperitoneal sarcoma (RPS): a report on 1,007 patients from the multi-institutional collaborative RPS working group. Ann Surg. (2016) 263:1002–9. 10.1097/SLA.0000000000001447 26727100

[7] Rajeev R Patel M Jayakrishnan TT Johnston FM Bedi M Charlson J. Retroperitoneal solitary fibrous tumor: surgery as first line therapy. Clin Sarcoma Res. (2015) 5:19 10.1186/s13569-015-0034-y 26322223 PMC4551387

[8] Luo P Wu Z Chen S Yang L Cai W Chen Y. Outcome of patients with primary retroperitoneal solitary fibrous sarcoma. Int. J Clin Oncol. (2020) 25:921–8 10.1007/s10147-020-01617-w 32140952

[9] Clavien PA Sanabria JR Strasberg SM. Proposed classification of complications of surgery with examples of utility in cholecystectomy. Surgery. (1992) 111:518–26. 1598671

[10] Cranshaw IM Gikas PD Fisher C Thway K Thomas JM Hayes AJ. Clinical outcomes of extra-thoracic solitary fibrous tumours. Eur J Surg Oncol. (2009) 35:994–8 10.1016/j.ejso.2009.02.015 19345055

[11] Jo VY Fletcher CD. WHO classification of soft tissue tumours: an update based on the 2013 (4th) edition. Pathology. (2014) 46:95–104 10.1097/PAT.0000000000000050 24378391

[12] O'Neill AC Tirumani SH Do WS Keraliya AR Hornick JL Shinagare AB. Metastatic patterns of solitary fibrous tumors: a single-institution experience. AJR Am J Roentgenol. (2017) 208:2–9. 10.2214/AJR.16.16662 27762594

[13] Kim MY Jeon S Do Choi S Nam KH Sunwoo JG Lee JH. A case of solitary fibrous tumor in the pelvis presenting massive hemorrhage during surgery. Obstet Gynecol Sci. (2015) 58:73–6 10.5468/ogs.2015.58.1.73 25629023 PMC4303757

[14] Wada Y Okano K Ando Y Uemura J Suto H Asano E. A solitary fibrous tumor in the pelvic cavity of a patient with Doege-Potter syndrome: a case report. Surg Case Rep. (2019) 5:60 10.1186/s40792-019-0617-6 30976927 PMC6459447

[15] Soda H Kainuma O Yamamoto H Nagata M Takiguchi N Ikeda A. Giant intrapelvic solitary fibrous tumor arising from mesorectum. Clin J Gastroenterol. (2010) 3:136–9 10.1007/s12328-010-0146-0 26190119

[16] Lahat G Anaya DA Wang X Tuvin D Lev D Pollock RE. Resectable well-differentiated vs. dedifferentiated liposarcomas: two different diseases possibly requiring different treatment approaches. Ann. Surg. Oncol. (2008) 15:1585–93 10.1245/s10434-007-9805-x 18398663

[17] Tseng WH Martinez SR Tamurian RM Chen SL Bold RJ Canter RJ. Contiguous organ resection is safe in patients with retroperitoneal sarcoma: an ACS-NSQIP analysis. J. Surg. Oncol. (2011) 103:390–4. 10.1002/jso.21849 21400521

[18] MacNeill AJ Gronchi A Miceli R Bonvalot S Swallow CJ Hohenberger P. Postoperative morbidity after radical resection of primary retroperitoneal sarcoma: a report from the transatlantic RPS working group. Ann. Surg. (2018) 267:959–64. 10.1097/SLA.0000000000002250 28394870

[19] Nessim C Raut CP Callegaro D Barretta F Miceli R Fairweather M. Postoperative morbidity after resection of recurrent retroperitoneal sarcoma: a report from the transatlantic Australasian RPS working group (TARPSWG). Ann. Surg. Oncol. (2021) 28:2705–14. 10.1245/s10434-020-09445-y 33389288

[20] Ejaz A Frank SM Spolverato G Kim Y Pawlik TM. Defining transfusion triggers and utilization of fresh frozen plasma and platelets among patients undergoing hepatopancreaticobiliary and colorectal surgery. Ann. Surg. (2015) 262:1079–85. 10.1097/SLA.0000000000001016 25985254

[21] Baecker A Liu X La Vecchia C Zhang ZF. Worldwide incidence of hepatocellular carcinoma cases attributable to major risk factors. Eur Cancer Prev. (2018) 27:205–12. 10.1097/CEJ.0000000000000428 29489473 PMC5876122

[22] Glance LG Dick AW Mukamel DB Fleming FJ Zollo RA Wissler R. Association between intraoperative blood transfusion and mortality and morbidity in patients undergoing non-cardiac surgery. Anesthesiology. (2011) 114:283–92. 10.1097/ALN.0b013e3182054d06 21239971

[23] Goldfarb Y Sorski L Benish M Levi B Melamed R Ben-Eliyahu S. Improving postoperative immune status and resistance to cancer metastasis: a combined perioperative approach of immunostimulation and prevention of excessive surgical stress responses. Ann. Surg. (2011) 253:798–810. 10.1097/SLA.0b013e318211d7b5 21475023

[24] Chang ED Lee EH Won YS Kim JM Suh KS Kim BK. Malignant solitary fibrous tumor of the pleura causing recurrent hypoglycemia; immunohistochemical stain of insulin-like growth factor i receptor in three cases. J. Korean Med. Sci. (2001) 16:220–4. 10.3346/jkms.2001.16.2.220 11306751 PMC3054728

[25] Saynak M Bayir-Angin G Kocak Z Oz-Puyan F Hayar M Cosar-Alas R. Recurrent solitary fibrous tumor of the pleura: significant response to radiotherapy. Med. Oncol. (2010) 27:45–8. 10.1007/s12032-009-9168-1 19165637

[26] Kawamura S Nakamura T Oya T Ishizawa S Sakai Y Tanaka T. Advanced malignant solitary fibrous tumor in pelvis responding to radiation therapy. Pathol. Int. (2007) 57:213–8. 10.1111/j.1440-1827.2007.02083.x 17316417

[27] Ghose A Guha G Kundu R Tew J Chaudhary R. CNS hemangiopericytoma: a systematic review of 523 patients. Am J Clin Oncol. (2017) 40:223–7. 10.1097/COC.0000000000000146 25350465

[28] Haas RL Walraven I Lecointe-Artzner E Scholten AN van Houdt WJ Griffin AM. Radiation therapy as sole management for solitary fibrous tumors (SFT): a retrospective study from the global sft initiative in collaboration with the sarcoma patients Euronet. Int J Radiat Oncol Biol Phys. (2018) 101:1226–33. 10.1016/j.ijrobp.2018.04.024 29859795

[29] Bonvalot S Gronchi A Le Péchoux C Swallow CJ Strauss D Meeus P. Preoperative radiotherapy plus surgery vs. surgery alone for patients with primary retroperitoneal sarcoma (EORTC-62092: STRASS): a multicentre, open-label, randomised, phase 3 trial. Lancet Oncol. (2020) 21:1366–77. 10.1016/S1470-2045(20)30446-0 32941794

[30] Yokoyama Y Hata K Kanazawa T Yamaguchi H Ishihara S Sunami E. Giant solitary fibrous tumor of the pelvis successfully treated with preoperative embolization and surgical resection: a case report. World J Sur. Oncol. (2015) 13:164 10.1186/s12957-015-0578-6 25924672 PMC4417519

[31] Velayati S Erinjeri JP Brody LA Ziv E Boas FE Brown KT. Safety and Efficacy of hepatic artery embolization in treating solitary fibrous tumor metastatic to the liver. Sarcoma. (2019) 2019:3060658 10.1155/2019/3060658 31565028 PMC6745165

[32] Demicco EG Park MS Araujo DM Fox PS Bassett RL Pollock RE. Solitary fibrous tumor: a clinicopathological study of 110 cases and proposed risk assessment model. Mod Pathol. (2012) 25:1298–306. 10.1038/modpathol.2012.83 22575866

